# Integrating care by implementation of bundled payments: results from a national survey on the experience of Dutch dietitians

**DOI:** 10.5334/ijic.1133

**Published:** 2013-12-18

**Authors:** J. Tol, I.C.S. Swinkels, J.N. Struijs, C. Veenhof, D.H de Bakker

**Affiliations:** NIVEL (Netherlands Institute for Health Services Research), The Netherlands; NIVEL, The Netherlands; RIVM (National Institute of Public Health and Environment), The Netherlands; NIVEL, The Netherlands; NIVEL, The Netherlands\TRANZO (Tilburg University, Scientific Centre for Transformation in Care and Welfare), P.O. Box 90153, 5000 LE Tilburg, The Netherlands

**Keywords:** dietetics, primary care, disease management, bundled payment, payment reform, integrated care

## Abstract

**Introduction:**

In the Netherlands, bundled payments were introduced as part of a strategy to redesign chronic care delivery. Under this strategy new entities of health care providers in primary care are negotiating with health insurers about the price for a bundle of services for several chronic conditions. This study evaluates the level of involvement of primary health care dietitians in these entities and the experienced advantages and disadvantages.

**Methods:**

In August 2011, a random sample of 800 Dutch dietitians were invited by email to complete an online questionnaire (net response rate 34%).

**Results:**

Two-thirds participated in a diabetes disease management programme, mostly for diabetes care, financed by bundled payments (*n*=130). Positive experiences of working in these programmes were an increase in: multidisciplinary collaboration (68%), efficiency of health care (40%) and transparency of health care quality (25%). Negative aspects were an increase in administrative tasks (61%), absence of payment for patients with comorbidity (38%) and concerns about substitution of care (32%).

**Discussion/conclusion:**

Attention is needed for payment of patients with co- or multi-morbidity within the bundled fee. Substitution of dietary care by other disciplines needs to be further examined since it may negatively affect the quality of treatment. Task delegation and substitution of care may require other competencies from dietitians. Further development of coaching and negotiation skills may help dietitians prepare for the future.

## Introduction

Many people suffer from chronic non-communicable diseases worldwide [1]. Unhealthy lifestyles, including unhealthy dietary patterns, are among the key risk factors for major chronic non-communicable diseases, such as cardiovascular diseases or diabetes [2]. Therefore, dietary treatment is an important aspect of the prevention and management of various chronic diseases. Increased prevalence of chronic diseases is predicted for the coming years. In line with this increase, there is a growing necessity for coordination of health care delivery for the chronically ill [3]. Consequently, health care providers and public policy makers have embraced the concept of disease management.

Disease management programmes were originally developed in the United States, and a range of countries have followed suit [4]. Some studies have shown that disease management programmes in general may contribute to better care for the chronically ill [5,6]. However, many countries are seeking ways to provide more effective and less-expensive care. In the Netherlands, a number of initiatives were introduced to improve the quality and reduce the costs of care for chronically ill patients [7]. The fragmentary nature of the funding of these initiatives, however, hindered the establishment of nationwide, long-term disease management programmes [8,9]. The Dutch minister of health therefore approved the implementation of a structural, bundled payment approach in 2010 for type 2 diabetes care, chronic obstructive pulmonary disease care and vascular risk management.

The Dutch bundled payment scheme aims to improve multidisciplinary collaboration and, consequently, to improve health care and the affordability of health care for patients with chronic diseases [10]. Under the bundled payment schemes, insurers now pay a single fee to a contracting entity, the care group, to cover all of the primary care needed to manage a chronic condition [7,10]. Care groups are often exclusively owned by general practitioners. The care group assumes both clinical and financial responsibility, often in a particular geographical region, on the basis of bundled payment contracts. A care group either subcontracts other care providers, such as general practitioners, practice nurses, dietitians and specialists or delivers the contracted care itself. The price for the bundle of services is freely negotiable by insurers and care groups, and the fees for the subcontracted care providers are likewise freely negotiable by the care group and providers [9]. Care services by care groups are provided in accordance with the Care Standards, which describe the care services and treatment activities (the ‘what’), but do not specify the providers (the ‘who’, ‘where’ and ‘how’) of those activities.

Experimentation with bundled payments was first introduced in the United States. Some of the plusses of bundled payments include their potential to improve coordination among multiple caregivers, flexibility in the delivery of care, incentive to reduce costs and one bill instead of many [11,12]. In the Netherlands, the first results from a national evaluation of care groups financed by bundled payments showed that this system improved the organisation and coordination of care and led to better collaboration among health care providers and greater adherence to care protocols. Negative results included dominance of the care group by general practitioners, large price variations in the bundled fee across care groups and the administrative burden [13].

Up to now, almost all studies examining the effect of the Dutch bundled payment approach have mainly focussed on the role of care groups and the effects of bundled payments on quality of care and health care expenditure [14]. Research specifically focusing on the perspectives of subcontracted caregivers is scarce. Only one study was aimed specifically at a subcontracted profession and included an explorative survey conducted among Dutch physical therapists. The study showed that physical therapists have little reason to participate in disease management programmes financed by bundled payments. Only a small percentage of patients in primary care physical therapy practices need chronic care such as diabetes care, chronic obstructive pulmonary disease care and vascular risk management. By contrast, for the profession of dietetics, the implementation of bundled payments may have a major impact, since dietitians frequently treat patients with diabetes, chronic obstructive pulmonary disease or patients with cardiovascular diseases and those at risk for cardiovascular diseases [15]. Prior to the implementation of bundled payments, dietitians were generally negative about the prospect and voiced concerns about substitution of care [16]. They feared, for example, that fewer patients would be referred for dietary advice due to competition from the practice nurse. Substitution of care could occur since the Care Standards include nutritional and dietary advice as an essential component in diabetes management, although the provider, price and volume of care are not specified [17]. This creates negotiation opportunities for dietitians, but it also poses a threat, as dietary advice can also be provided by other competent care providers, such as the general practitioner or practice nurse. A dietitian's participation in disease management programmes is therefore not an absolute given. Similarly, this is also the case in the United States [18] and Canada [19].

In 2011, diabetes care groups covered almost all regions in the Netherlands and almost 90% of diabetes care groups had contracted one or more dietitians [20]. A survey of dietitians, however, found that the percentage involved in a care group was considerably lower (66% in September 2010), and many were not even planning to get involved [21]. This raises questions about dietitians’ perceptions of bundled payments. A limitation of that survey was the relatively small sample of dietitians who filled out the questionnaire (response rate 17%), plus the fact that the results were not specified to dietitians working in disease management programmes financed by bundled payments. Therefore, the current study aims to explore dietitians’ experience of working in disease management programmes financed by bundled payments. Knowledge about this topic should provide insight for policymakers and dietitians about the pros and cons of a bundled payment scheme in order to operate according to the principles of disease management. Accordingly, an international audience can benefit from the lessons learned, since different payment methods for disease management programmes are frequently under discussion [11]. See Box 1 for more information about the organisation and payment system of dietetics in the Netherlands.

To summarise, the research questions of this exploratory study are (1) To what extent are Dutch primary health care dietitians involved in disease management programmes financed through bundled payments? (2) What are the experiences and opinions of Dutch primary health care dietitians with regard to working in disease management programmes financed through bundled payments?

## Subjects and methods

### Participants

For the purpose of this explorative study, 800 dietitians were randomly selected from a membership list containing all e-mail addresses of the members of the Dutch Dietetic Association. The 800 dietitians represented 65% of all primary care dietitians [23]. Only dietitians working in primary health care were eligible to participate. Dietitians who were not actively practising in the Netherlands were excluded.

### Questionnaire

Data were collected through an online survey in August 2011. The participants received an e-mail with a covering letter describing the aims of the study and containing a personal html link with log-in password in order to complete the questionnaire online. Non-respondents were sent a reminder e-mail after three weeks, and a second reminder after a further three weeks. To increase the response, three raffle-type draws for a 50-euro gift voucher were held.

The questionnaire was based on a previously designed questionnaire measuring the involvement of Dutch physical therapists in disease management programmes financed by bundled payments. The latter questionnaire had been based on a literature search and semi-structured interviews with experts in the field of bundled payments. For the current questionnaire, topics were extended and adjusted to include issues that were relevant for the dietetic profession. The authors of this study developed the questionnaire. Subsequently, the questionnaire was reviewed by experts of the Dutch Dietetic Association as well as the same bundled payment experts who had previously been involved in the development of the questionnaire for physical therapists.

The first part of the questionnaire collected general information on respondents’ age, gender, years of experience, work setting and region of employment. The second part of the questionnaire collected information on dietitians’ involvement in disease management programmes financed through bundled payments, and their experiences and opinions with regard to working in programmes of this nature (see Table 1).

### Statistical analyses

We performed descriptive statistical analyses to investigate the involvement, experiences and opinions of dietitians regarding disease management programmes financed by bundled payments. Data on non-respondents were not available. However, to investigate the generalisability of the results, statistical analyses were conducted to test for a significant difference (*p*<0.05) between the general characteristics of the respondents compared to the primary health care dietitians who were member of the Dutch Dietetic Association. An independent samples’ *t*-test was used to examine mean differences in age and number of years of professional experience between the two groups. Chi-squared tests were used to determine if significant differences in gender and regional distribution existed between the two groups. Missing data were not included; the data were analysed using STATA version 11.

## Results

### Response and general information

**Figure 1.**
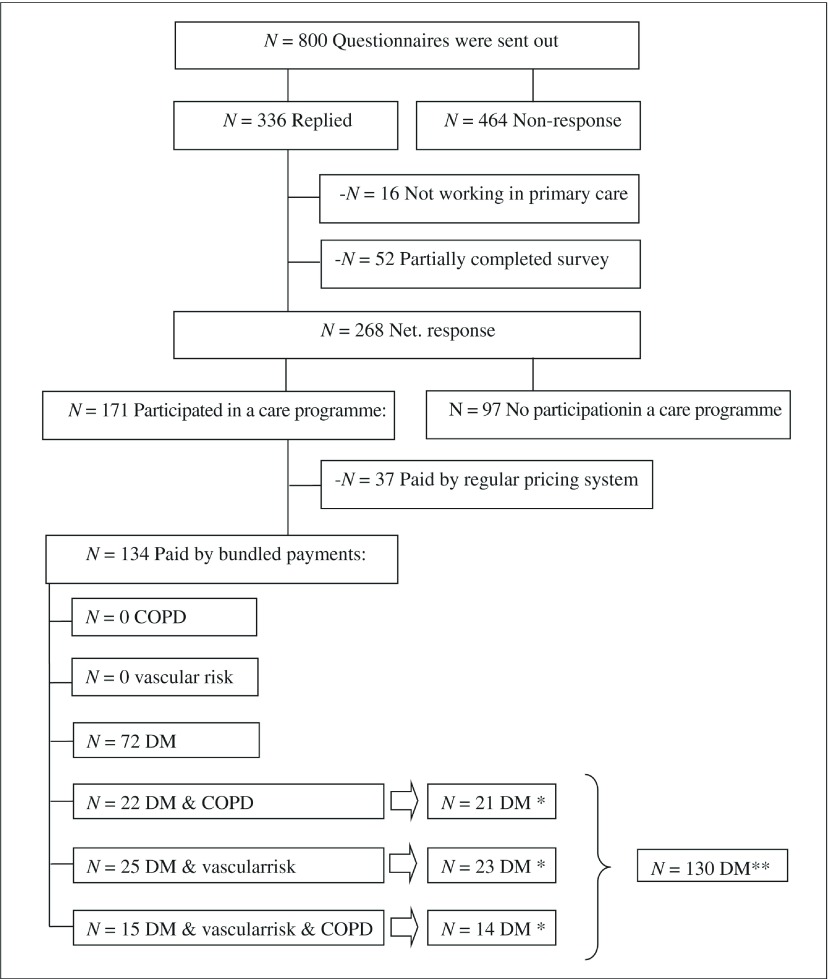
Response and involvement in disease management programmes. *The dietitians who participated in more than one disease management programme financed by the system of bundled payments were asked to complete the questionnaire regarding the care group where they treated most of their patients. Most patients were treated in a diabetes care programme. **Results were shown for dietitians who participated in a diabetes disease management programme financed by bundled payments.

Of the 800 dietitians surveyed, 336 (42%) dietitians responded, of whom 320 were eligible to participate; 16 respondents did not work as a dietitian in primary health care. A total of 268 (net response rate 34%) dietitians completed the entire questionnaire (see Figure 1). The majority worked in private practice (69%). The respondents were representative to all members of the Dutch Dietetic Association for years of work experience (average 16 years, *p*=0.96), gender (98% were female, *p*=0.82) and region of residence (*p*=0.08). However, the respondents were significantly older compared to all members of the Dutch Dietetic Association, with a mean age of 42.5 versus 40.0 (*p*<0.01).

### Involvement in disease management programmes financed by bundled payments

Two-third of the 268 respondents participated in at least one of the three disease management programmes (*n*=171) (see Figure 1). Excluded from this study were results from dietitians who participated in a disease management programme where dietetic care was exclusively financed by the ‘regular’ pricing system (*n*=37), i.e. dietitians claimed for the delivered care directly from the insurance companies. The majority of dietitians participated in a disease management programme were financed by bundled payment schemes, i.e. dietitians were paid by the care group or a combination of the care group and the ‘regular’ pricing system (*n*=134). Almost half of the dietitians participated in more than one disease management programme financed by bundled payment schemes (46% of 134). Overall, most of their patients were treated in a disease management programme for diabetes type 2 (*n*= 130). Therefore, the results for vascular risk management and chronic obstructive pulmonary disease care were not taken into account.

Almost all dietitians who participated in a bundled payment disease management programme on diabetes were subcontracted by the care group (95% of 130). Most of the time, the dietitians in a region were individually contracted (67% of 124). Some dietitians reported that care groups limited the number of dietitians eligible to participate (10% of 124).

The main reported reasons for not participating in a disease management programme were (1) a lack of initiatives in the region (32% of 97) and (2) not being approached by a care group (27% of 97). Only a limited number of dietitians (12% of 97) were unable to participate because the care group did not intend to subcontract a dietitian.

The main tasks of the dietitian in a diabetes disease management programme were to provide individual medical nutrition therapy (91%) and individual education (35%). About a third of the dietitians were also contracted to coach the practice nurse regarding dietary counselling. Less than five percent of the dietitians were involved in management and/or governance tasks.

### Advantages

**Figure 2.**
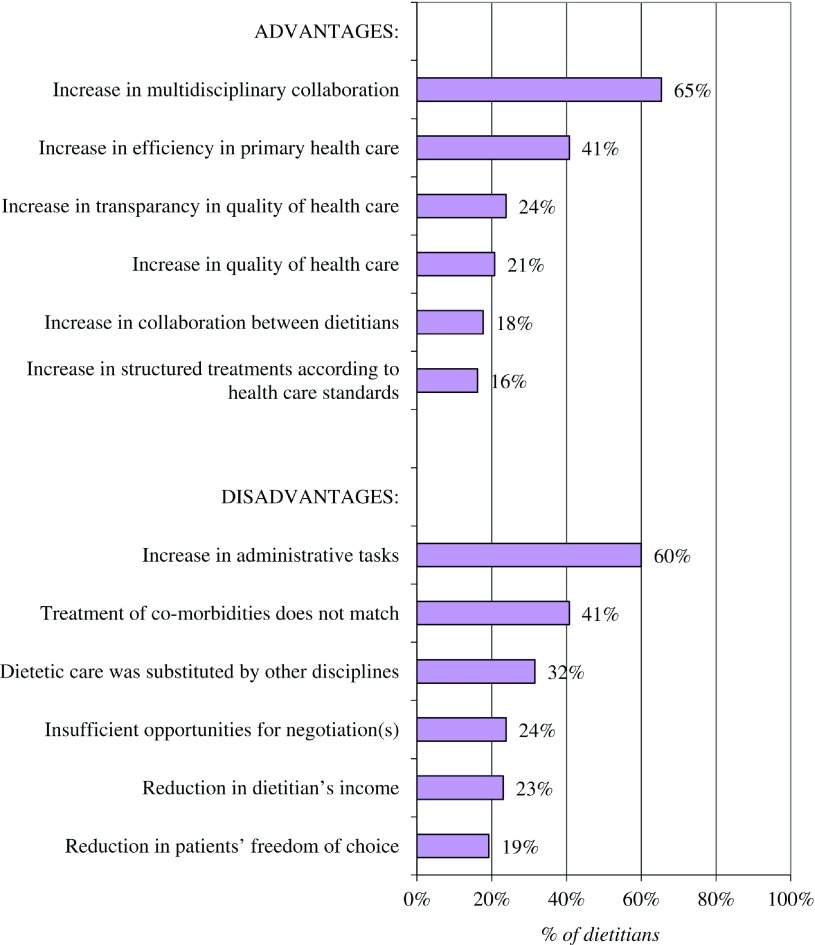
Six most frequently cited advantages and disadvantages of bundled payments (maximum of three answers per dietitian, *n*=130).

An increase in multidisciplinary collaboration (65% of 130) was one of the three most frequently mentioned advantages of working in a bundled payment disease management programme. For example, one out of three dietitians (*n*=47) mentioned that the relationship with the general practitioner had changed, usually in a positive manner. Three frequently cited changes were easier access to the practice nurse (70.2% of 47), increased contact frequency initiated by the practice nurse (66% of 47) and increased number of meetings with the general practitioner about patients’ treatment (49% of 47). The second and third most frequently mentioned advantages were more efficiency in primary healthcare (41%) and greater transparency of health care quality (24%) (see Figure 2).

### Disadvantages

The most frequently mentioned disadvantage of the bundled payment scheme was an increase in administrative tasks (60%). For example, 60% of dietitians had to cope with double registration information in their usual electronic health record and in the electronic health record used by the care group. The majority of dietitians registered double information for personal details (68% of 78), appointments (65% of 78), measurements (63% of 78) and payments (59% of 78). The second and third most frequently mentioned disadvantages were a lack of payment for patients with co- or multi-morbidity (41%), and that dietetic care was substituted by other disciplines (32%). The majority of dietitians (fully) believed that substitution of dietetic care was happening (55%), though 31% did not have an opinion about this issue.

## Discussion

Almost two years after the introduction of the bundled payment scheme, two-thirds of Dutch primary health care dietitians participated in a disease management programme. The majority were subcontracted by a care group to deliver medical nutrition therapy in a diabetes disease management programme financed by bundled payments. Both positive and negative aspects of the bundled payment scheme were reported by the dietitians.

Regarding the involvement of dietitians in disease management programmes, the results seem comparable with the findings of a study one year earlier [21]. The absence of an increase was not related to a lack of willingness among dietitians to participate. The most frequently mentioned reason for not participating in a care group was a lack of initiatives in the region. However, in 2011, diabetes care groups were represented in all regions in the Netherlands [20]. Comparing the regional distribution of dietitians with the regional coverage of diabetes disease management programmes (results not shown), it seems unlikely that there were no programmes in any respondent's region of residence. Therefore, the awareness of the existence of care groups in the region should be promoted among relatively small professional health care disciplines, in this case dietetics. Another frequently mentioned reason for not participating was not being approached by a care group. However, dietitians themselves could take the initiative in this respect. Few dietitians were unable to participate because the care group did not intend to include a dietitian. Therefore, watchfulness is needed, since excluding dietitians from care groups may result in decreased access to dietetic care for patients within diabetes care groups, with limited freedom of choice as a result [25].

Dietitians who participated in a disease management programme on diabetes most frequently reported increased multidisciplinary collaboration as an important advantage of bundled payments. This was consistent with results from the national evaluation of Dutch care groups [13]. Although greater efficiency of health care and transparency of health care quality are among the most frequently reported advantages of care groups, only a minority of dietitians mentioned these as an advantage. Therefore, improvements would seem necessary. A lack of transparency in the quality of delivered care is a major problem for dietitians, as the care services provided by the dietitian can be substituted by other disciplines in the bundled payment model. Transparency can be improved in the future by promoting the development and implementation of electronic health records. For example, registered data on the dates and time of treatment visits, treatment process and performance indicators could be used for negotiations with care groups. The most frequently mentioned negative aspect of the bundled payment scheme was an increase in administrative tasks as a consequence of the necessity of registering the same data in multiple information technology applications. All providers register data in their own electronic health records but are also obliged to register these data in the care group's electronic health records. As a consequence of the lack of an adequate integration of the information technology applications, the administrative burden of subcontracted caregivers has increased. However, these record-keeping obligations have also led to a reported advantage, namely increased transparency of the quality of care delivered. Therefore, the integration of the different electronic health records needs to be fostered in order to support the electronic registration and payment system for patient care within a care group.

The second most important disadvantage was a lack of payment for patients with co- or multi-morbidity within the bundled fee. This problem occurs as the bundled payment scheme has a single-disease focus, meaning that only care services for diabetes were included in the bundled fee and no services related to coexisting conditions. This is despite the fact that 90% of the patients with diabetes who visit a dietitian have coexisting conditions [26]. Working with single-disease bundled payments for specific chronic conditions might result in a compartmentalised health care delivery system for patients with co- or multi-morbidity. A global payment approach could be a solution to this problem. Recently, the Dutch Minister of Health announced new payment reforms which might include this global payment approach [27]. Under the proposed reforms, care groups would receive a specified amount of money per enrolled resident based on the characteristics of the population. In principle, it will address all required health care for an assigned population, financed by a single amount per assigned citizen. Bundled payments can therefore be seen as an intermediate step towards the delivery of real integrated care with a global payment approach as the ultimate goal [25].

Another important disadvantage for the dietitian was that dietetic care was substituted by other disciplines, such as the practice nurse. The majority of dietitians (fully) believed that substitution of dietetic care was taken place. An evaluation study by Van Dijk et al. showed similar results for substitution of dietetic health care [28]. In general, task delegation and substitution of care were encouraged by care groups and were aimed at reducing health care costs and improving the efficiency of diabetic care [29]. Task delegation and substitution of care may have consequences for dietitians. Negative effects may include a reduction in their income. Positive effects may include an involvement in disease management programmes. These may consist of coaching and training the practice nurse to give general dietary advice, and giving dietary advice to patients with more complex health problems. Task delegation and substitution of care may require other competencies from dietitians, such as coaching skills and negotiation skills to obtain a proper contract. Dietitians could prepare themselves for the future by developing these skills. Recently, a nutrition care module was published which provides insight into the different types of nutritional care and the requirements for the delivery of adequate nutritional care by caregivers with the right competencies [25]. Dietitians can actively use this module for negotiations, supplementary to the Care Standards. Consequently, the question remains whether task delegation and substitution of dietetic care may negatively affect the quality of treatment. There is no strong evidence demonstrating that treatment by a dietitian achieves better outcomes than treatment by practice nurses [30,31]. Therefore, research is needed to evaluate the effectiveness of dietetic treatment and the impact of substitution of dietary counselling by other disciplines.

A strength of the study was the accessibility of the questionnaire, enabled by the fact that the majority of Dutch primary health care dietitians (65% of total) were approached by e-mail with a covering letter and a personal html link with a view to filling out the questionnaire online. Another strength was the response rate obtained. Even though the response rate seems relatively low, this study surveyed 20% of all Dutch primary care dietitians. In addition, the response rate was twice as high as compared to a survey conducted among dietitians [21] and was comparable with the response rates of a survey conducted among physical therapists. A limitation of our study was the establishment of the respondent's representativeness. No information was available on non-respondents. It is possible that dietitians without experience of bundled payments or of care groups may not have felt drawn to participating. We do not believe that this has led to an overestimation of the number of dietitians participating in care programmes, since the results were comparable to those from one year earlier [21]. In addition, the respondents were representative for number of years worked, gender and regional distribution compared to the members of the Dutch Dietetics Association.

## Conclusion

Almost two years after the introduction of the bundled payment scheme, two-thirds of Dutch primary health care dietitians participated in a disease management programme. The majority were subcontracted to deliver medical nutrition therapy in a disease management programme for diabetes type 2 financed by bundled payments. Both positive and negative aspects were reported. Positive aspects were an increase in: multidisciplinary collaboration, efficiency of health care and transparency in quality of care delivered. Negative reported aspects were: an increase in administrative tasks as a consequence of double reporting, absence of payment for patients with co- and multi-morbidity and concerns about care substitution. The effect of substitution of dietary counselling by other disciplines needs to be further examined since it may negatively affect the quality of treatment. Furthermore, task delegation and substitution of care may require other competencies from dietitians. For this reason, they could prepare themselves for the future by developing their coaching and negotiation skills.

## Figures and Tables

**Box 1.**
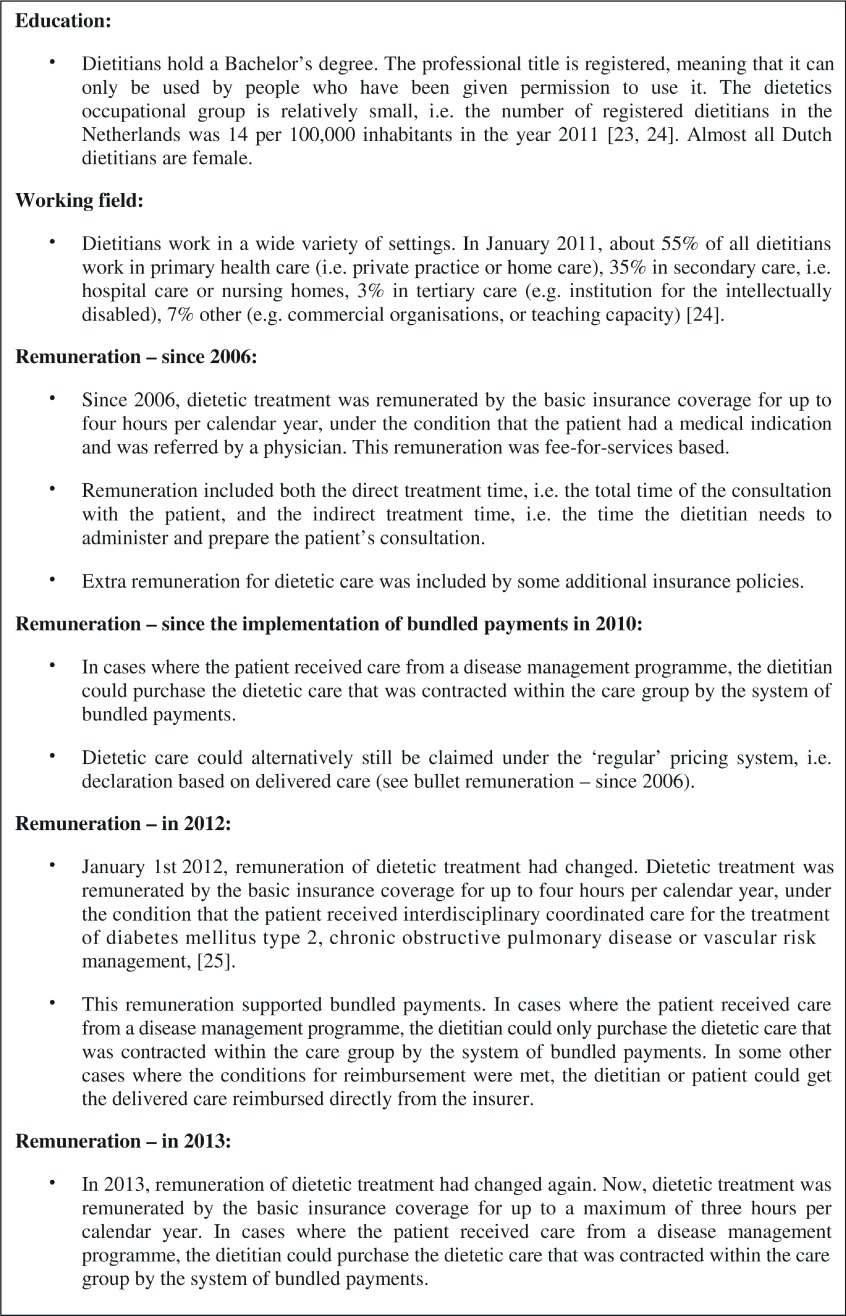
General description of education, working field and remuneration of dietitians in the Netherlands.

**Table 1.**
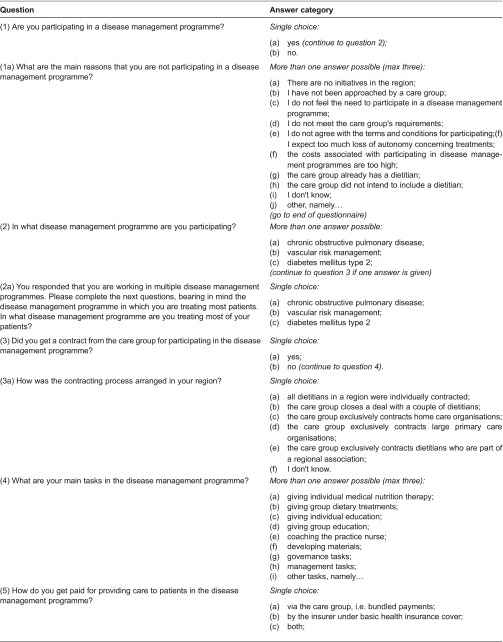

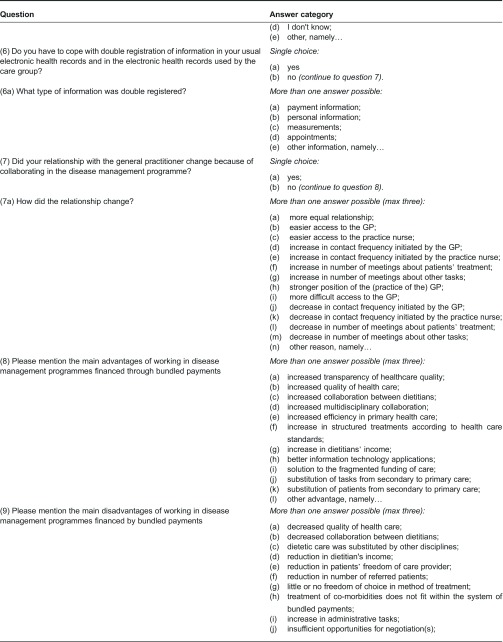

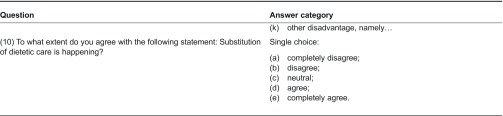
Content of the questionnaire

Abbreviations: GP, general practitioner.
